# Hit-and-run programming of therapeutic cytoreagents using mRNA nanocarriers

**DOI:** 10.1038/s41467-017-00505-8

**Published:** 2017-08-30

**Authors:** H. F. Moffett, M. E. Coon, S. Radtke, S. B. Stephan, L. McKnight, A. Lambert, B. L. Stoddard, H. P. Kiem, M. T. Stephan

**Affiliations:** 1 Clinical Research Division, Fred Hutchinson Cancer Research Center, Seattle, WA 98109 USA; 2 Division of Basic Sciences, Fred Hutchinson Cancer Research Center, Seattle, WA 98109 USA; 3 Department of Medicine, Division of Medical Oncology, University of Washington, Seattle, WA 98109 USA; 4 Department of Bioengineering and Molecular Engineering & Sciences Institute, University of Washington, Seattle, WA 98105 USA

## Abstract

Therapies based on immune cells have been applied for diseases ranging from cancer to diabetes. However, the viral and electroporation methods used to create cytoreagents are complex and expensive. Consequently, we develop targeted mRNA nanocarriers that are simply mixed with cells to reprogram them via transient expression. Here, we describe three examples to establish that the approach is simple and generalizable. First, we demonstrate that nanocarriers delivering mRNA encoding a genome-editing agent can efficiently knock-out selected genes in anti-cancer T-cells. Second, we imprint a long-lived phenotype exhibiting improved antitumor activities into T-cells by transfecting them with mRNAs that encode a key transcription factor of memory formation. Third, we show how mRNA nanocarriers can program hematopoietic stem cells with improved self-renewal properties. The simplicity of the approach contrasts with the complex protocols currently used to program therapeutic cells, so our methods will likely facilitate manufacturing of cytoreagents.

## Abstract (web summary)

Current widely used viral and electroporation methods for creating therapeutic cell-based products are complex and expensive. Here, the authors develop targeted mRNA nanocarriers that can transiently program gene expression by simply mixing them with cells, to improve their therapeutic potential.

## Introduction

Therapeutic methods based on immune cells have experienced a substantial metamorphosis from interventions involving straightforward blood transfusions and bone marrow transplants into a nascent healthcare industry. Currently, over 500 companies are involved in the development and commercialization of cell-based therapeutic products^1^, and hematopoietic stem cell (HSC) transplants have evolved into the standard-of-care for treating leukemia and other bone and blood cancers (with over one million transplants performed worldwide to date^2^). But also, different sorts of cell therapy products are undergoing clinical evaluation for treating a variety of diseases, including tissue degeneration, chronic inflammation, autoimmunity, genetic disorders, cancer, and infections^3–8^. It has become possible to focus immune responses towards these diseases by genetically engineering T-cells to express targeted chimeric antigen receptors (CARs) or T cell receptors (TCRs), and this approach has presented positive clinical responses in cancer patients who have no other curative options^9, 10^. Thanks to a strong clinical presence, the expanding array of cell therapy products has catalyzed the field of cellular bioengineering with the goal of maximizing the therapeutic performance of these cytoreagents in patients^11, 12^.

Some gene therapy applications require chronic expression systems that stably integrate the engineered transgene into the patient’s DNA. One example is the expression of cancer-specific receptor genes by T-cells, which converts them into ‘living drugs’ that can increase in number while they serially destroy tumor cells^9, 10^. Another is the introduction of gamma-globin genes into transplanted HSCs as a way to reverse beta thalassemia^13^. Despite the time and cost required for their production, as well as restrictions on the size and number of genes that they can package, viral vectors are currently the most effective means to stably express these transgenes^14, 15^.

It is also possible to elicit phenotypic changes via transient expression of macromolecules, designed to accomplish “hit-and-run” genetic programming. In most of these kinds of applications, permanent expression of the therapeutic transgene is undesirable and potentially dangerous^16^. Examples include the use of transcription factors to control cell differentiation^17, 18^, and the expression of sequence-specific nucleases to engineer genomes^19^.

Although there is a growing number of applications where transient gene therapy could improve the curative potential of engineered cells, currently available methods (which, like the chronic expression methods described above, are mostly based on viral vectors) are complicated by the time and expense involved in the elaborate protocols required for their implementation^20^. Electroporation is an alternative transfection method, but physical permeabilization of plasma membranes compromises cell viability, which means these approaches are not suited for scale-up applications. Besides, like virus-based methods, electroporation cannot selectively transfect specific cell types from a heterogeneous pool, so it must be preceded by a cell purification process.

**Fig. 1:**
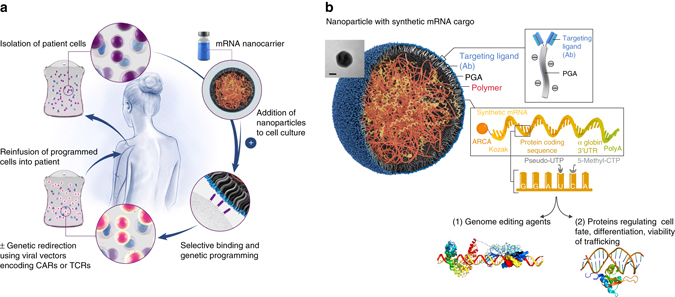
Creating mRNA nanoparticles to program therapeutic T-cells. **a** Schematic explaining how cultured T-cells can be programmed to express therapeutically relevant transgenes carried by polymeric nanoparticles (NPs). These particles are coated with ligands that target them to specific cell types, enabling them to introduce their mRNA cargoes and cause the targeted cells to express selected proteins (like transcription factors or genome-editing agents). **b** Design of targeted mRNA-carrying NPs. The inset shows a transmission electron micrograph of a representative NP; *scale bar*, 50 nm. Also depicted is the synthetic mRNA encapsulated in the NP, which is engineered to encode therapeutically relevant proteins

Here, we describe a nanoreagent that, via a comparatively simple process, produces transient gene expression in cultured cells. We demonstrate that an appropriately designed messenger RNA (mRNA) nanocarrier can accomplish dose-controlled delivery of functional macromolecules to lymphocytes or HSCs simply by mixing the reagent with the cells in vitro (Fig. 1a). These nanoparticles (NPs) can be designed to target particular cell subtypes and, upon binding to them, stimulate receptor-mediated endocytosis, thereby introducing the synthetic mRNA they carry which the cells can now express. Because nuclear transport and transcription of the transgene are not required, this process is fast and efficient. Here, we illustrate in three examples how this new platform can be implemented to manufacture effective cell products for clinical use.

In the first case, we used mRNA nanocarriers to edit the genome of T-cells and established that targeted delivery of mRNA encoding a rare-cleaving megaTAL nuclease^21^ into lymphocytes can efficiently disrupt their expression of T cell receptors. In the second application, we transiently expressed Foxo1 to reprogram the differentiation of effector cells into functionally competent memory cells^22, 23^. Our results demonstrate that engineered NPs can bias CAR-T-cells toward a central memory phenotype.

To assure that our transient gene delivery platform can easily be integrated into existing protocols for manufacturing other therapeutic cell types, we engineered our third example in HSCs. For many decades, these have been successfully used for treating hematological and immune diseases^2^. However, their limited number, especially when isolated from umbilical cord, prevents broader application of HSC-based therapies. Attempts to propagate these cells in vitro have generally failed, primarily because self-renewal and in vivo regenerative capacity are rapidly lost in culture^24, 25^. We used our nanotechnology platform to deliver transgenes into cultured HSCs, and demonstrated that transient expression of a key “stemness” regulator (Musashi-2 protein^26^) increases their capacity for self-renewal and maintains therapeutically desirable properties in the stem cells.

The most significant benefit of our system is its simplicity in achieving genetic modifications of therapeutic cells at a clinical scale: all that is required is mixing the appropriate NP reagent with the cultured cells. Our approach patently contrasts with those currently used to transiently deliver genetic materials, which are less effective and involve many expensive and proprietary procedures that limit their availability. Beyond the T-cells and HSCs tested in our experiments, the technology described here could be adapted to improve the curative potentials of other cell types used in the clinic to treat disease (e.g., natural killer cells, regulatory T-cells, dendritic cells, or mesenchymal stem cells) without increasing handling time, risk, or complexity.

## Results

### Designing mRNA nanocarriers to choreograph gene expression

To create a reagent that can genetically modify primary T lymphocytes (which are notoriously refractory to non-viral transfection methods) simply by contact, we bioengineered polymeric NPs comprised of four functional components (Fig. 1b): (i) surface-anchored targeting ligands that selectively bind the NPs to T-cells and initiate rapid receptor-induced endocytosis to internalize them. In our experiments we used anti-CD3 and anti-CD8 antibodies; (ii) a negatively charged coating that shields the NPs to minimize off-target binding by reducing the surface charge of the NPs. Because it is already widely used in drug delivery platforms, we selected polyglutamic acid (PGA) to accomplish this; (iii) a carrier matrix that condenses and protects the nucleic acids from enzymatic degradation while they are in the endosome, but releases them once the particles are transported into the cytoplasm, thereby enabling transcription of the encoded protein. For this, we used a biodegradable poly(β-amino ester) (PBAE) polymer formulation that has a half-life between 1 and 7 h in aqueous conditions; and (iv) nucleic acids that are encapsulated within the carrier and produce gene editing or transient expression of proteins that can permanently alter the phenotype of the T-cell. mRNA is an ideal platform for transient therapeutic protein expression, because it has no potential for genomic integration and does not require nuclear localization for expression. However, unmodified mRNA can activate intracellular toll-like receptors, limiting protein expression and leading to toxicity^27^. To improve the stability and reduce the immunogenic potential of the mRNA we deliver, we used synthetic versions that incorporate modified nucleotides. For example, substitution of uridine and cytidine with the engineered bases pseudouridine and 5-methyl-cytidine synergistically blocks identification by innate pattern recognition receptors and increases mRNA translation^28^.

The NPs were manufactured utilizing a two-step, charge-driven self-assembly process. First, the synthetic mRNA was complexed with a positively-charged PBAE polymer, which condenses the mRNA into nano-sized complexes (Supplementary Fig. 1a, b). This step was followed by the addition of antibody-functionalized PGA, which shields the positive charge of the PBAE-mRNA particles and confers lymphocyte-targeting. The resulting mRNA nanocarriers had a size of 109.6 ± SE/26.6 nm and an almost neutral surface charge (1.1 ± SE/5.3 mV zeta potential, Supplementary Fig. 1c).

### T-cell viability following mRNA nanocarrier transfection

**Fig. 2:**
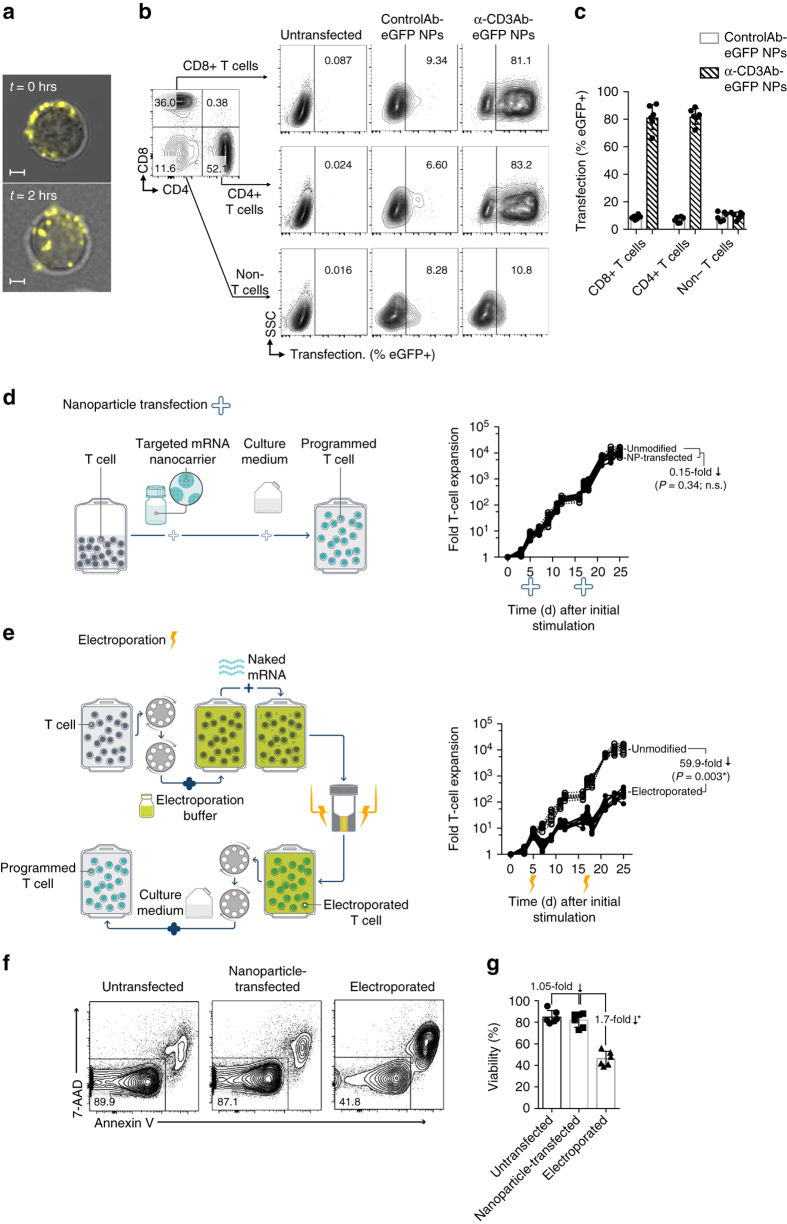
mRNA nanoparticle transfection choreographs robust transgene expression by lymphocytes. **a** Primary T-cells were mixed with CD3-targeted polymeric nanoparticles (NPs) carrying Cy5-labeled mRNA. Confocal microscopy establishes that these particles are rapidly internalized from the cell surface. The images are representative of 15 randomly chosen fields. *Scale bars*, 2 μm. **b** Flow cytometry of preactivated PBMCs 24 h after incubation with CD3-targeted or isotype control antibody-targeted nanoparticles bearing eGFP-encoding mRNA. **c** *Bar graph* summarizing transfection efficiencies from three independent experiments conducted in duplicate. **d**, **e** Comparison of the effects electroporation and NP gene delivery have on cell expansion. *Left panels* show the workflow for transfection with NPs (*top*) and electroporation (*bottom*). *Right panels* show the -fold expansion of PBMC cultures from three independent donors treated with stimulatory beads on days 0 and 12. Matched cultures from each donor were not treated, or transfected using CD3/CD28-targeted NPs (**d**, *right*) or electroporation (**e**, *right*) on days 5 and 17. Every line represents one donor and each dot reflects the -fold T-cell expansion. Pairwise differences between groups were analyzed with the unpaired, two-tailed Student’s *t* test; n.s., non-significant; *, significant, *n* = 3). **f** Relative viability of NP-transfected and electroporated T-cells. Samples of 2 × 10^6^ activated T-cells per condition were untreated, transfected with NPs, or electroporated. 18 h after treatment, cells were labeled with fluorescent dyes to assess viability. Results from three separate experiments conducted in duplicate are summarized in the *bar graph* shown in **g**. Statistical analysis between groups was performed using the unpaired, two-tailed Student’s *t* Test. *P < 0.0001

Our goal is to streamline the manufacture of cell-based therapies, so we first tested whether simply adding targeted mRNA nanocarriers to an established culture of human lymphocytes is sufficient to choreograph robust transfection in them. We found that when CD3-targeted NPs carrying mRNA encoding a reporter (enhanced green fluorescent protein, eGFP) are incubated with these cells, they not only bind to them but also stimulate receptor-mediated endocytosis, providing entry for the genes they carry (Fig. 2a). Following a single NP application (NP:T cell ratio = 2 × 10^4^:1), we routinely transfected >80% of these primary T-cells (Fig. 2b, c), with transgene expression observed as early as 5 h post-transfection (Supplementary Fig. 2a–c). Thus, this process is fast and efficient. Importantly, it was not necessary to prepare mRNA NPs freshly for each application—it was possible to lyophilize them before use with no change in properties or efficacy (Supplementary Fig. 2d). We also found that CD3-targeted nanoparticles selectively bind T lymphocytes, as their interactions with off-target cells were low (Fig. 2b, c; Supplementary Fig. 3). T cell proliferation did not impair NP-uptake/transfection, as (i) gene transfer into naive versus effector T-cells was comparable (Supplementary Fig. 4a, b), and (ii) addition of nanoparticles to stimulated T-cells at the peak of their expansion (day 17) yielded similar transfection relative to adding nanoparticles to freshly stimulated cells (day 5; Supplementary Fig. 4c).

We next assessed the impact of targeted mRNA^−^carrying NPs on T-cell expansion. Because malignancies often progress quickly, it is important that engineered T-cells can be expanded to clinically relevant scales equally quickly. One widely used approach to multiply polyclonal lymphocytes is to incubate them with beads that are coated with antibodies against TCR/CD3 and co-stimulatory CD28 receptors. We found that even repeated transfections with CD3-targeted NPs did not interfere with T-cell expansion stimulated by these coated beads (Fig. 2d). These results contrast sharply with T-cell electroporation, which we tested side-by-side. Not only did electroporation add complex handling steps (Fig. 2e), it compromised viability of the lymphocytes (Fig. 2f, g) and reduced their yield by 60-fold (Fig. 2e, right panel).

### Nanoparticle methods integrate into CAR-T cell manufacture

**Fig. 3:**
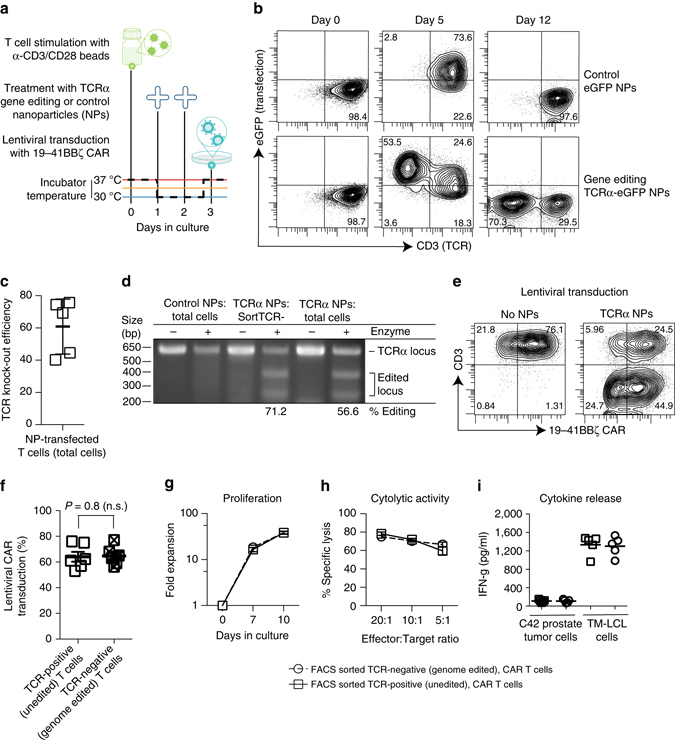
Nanoparticles can knockout T cell receptors in CAR-programmed lymphocytes. **a** Integration of nanoparticle (NP) transfection into normal manufacturing of CAR-T-cells. After stimulation with anti-CD3/CD28-coated beads (day 0), CD8-targeted mRNA NPs were introduced on days 1 and 2, then lentiviral transduction with a vector encoding the leukemia-specific 19-41BBz CAR was performed on day 3. We added either NPs carrying mRNAs encoding megaTAL nuclease plus eGFP, or control particles loaded with eGFP mRNA alone. **b** Flow cytometry of NP transfection efficiencies (based on eGFP signals) correlated with surface expression levels of TCRs (based on CD3 signals) by T-cells following NP treatments. **c** Summary plot showing editing efficiency as measured by loss of CD3 surface expression at day 14 (*n* = 6). **d** Surveyor assay confirming TCRα chain gene locus disruption. **e** Flow cytometry of lentiviral transduction in genome-edited versus control T-cells. **f** *Bar graph* showing mean viral transductions and SE of three independent experiments conducted in duplicate; n.s., not significant **g**, **h** Proliferation and cytolytic activity of TCR+ (FACS sorted TCR-positive, unedited 19-41BBz CAR-T-cells) and TCR- (FACS sorted TCR-negative, genome edited) 19-41BBz CAR-T-cells. To measure proliferation, T-cells were co-cultured on irradiated TM-LCL leukemia cells. Cytolytic assays were performed with CD19-expressing K562 target cells. **i** T cell IFN-γ release was measured with ELISA 48 h after stimulation on CD19+ TM-LCL leukemia cells or control LNCaP C4-2 prostate adenocarcinoma cells. Data from two experiments run in triplicate are shown

To test our approach in a clinically relevant application, we incorporated NP-mediated mRNA transfection methods into the manufacture of leukemia-specific 19-41BBζ CAR T-cells (Fig. 3a). CD19-targeted receptors are the most investigated CAR-T cell product today, with nearly 30 ongoing clinical trials internationally^29^. Our ability to perform genome engineering offers the potential to improve the safety and efficacy of CAR-T-cells. For example, we can inhibit expression of endogenous TCRs to avoid graft-versus-host disease, or selectively delete immune checkpoint genes in these cells to strengthen their anti-cancer activity in the suppressive tumor milieu^30, 31^. Here, we tested the ability of NPs to deliver gene-editing agents by preparing particles carrying mRNA encoding megaTAL nuclease, which targets the constant region (TRAC) of the TCR alpha gene. Taking advantage of the flexibility offered by our NP formulation methods, we included mRNA for the DNA repair endonuclease TREX2 to improve knockout efficiency^32^, along with eGFP mRNA so we could track transfection. Control particles were loaded with eGFP mRNA only. We found that, in contrast to eGFP-transfection (which did not impact TCR expression; Fig. 3b, top row), the addition of TCRα-megaTAL-carrying particles to the T-cell culture efficiently disrupted TCR expression by day 5, an effect that was maintained after loss of the mRNA by day 12 (Fig. 3b, bottom row). Average TCR knockout efficiency was 60.8% ( ± SE/17.7%; Fig. 3c), which corresponds with the percentage of indel frequencies (a measure of targeting efficiency) determined using the Surveyor assay (Fig. 3d). Importantly, the presence of mRNA-carrying NPs did not affect virus-mediated gene transfer of the tumor-specific CAR, as we achieved equal transduction efficiencies with a lentiviral vector encoding 19-41BBζ CAR in NP-transfected and non-transfected T-cells (Fig. 3e, f). Following NP-mediated genome editing and lentiviral transduction, CAR-programmed T-cells fully maintained their capacities to proliferate, secrete cytokines, and eliminate leukemia target cells (Fig. 3g–i). In summary, these findings establish that lymphocyte-targeted mRNA nanocarriers can mediate efficient genome editing of CAR-T-cells without compromising their function.

### Nanoparticle programming for Foxo1 enhances CAR-T-cells

**Fig. 4:**
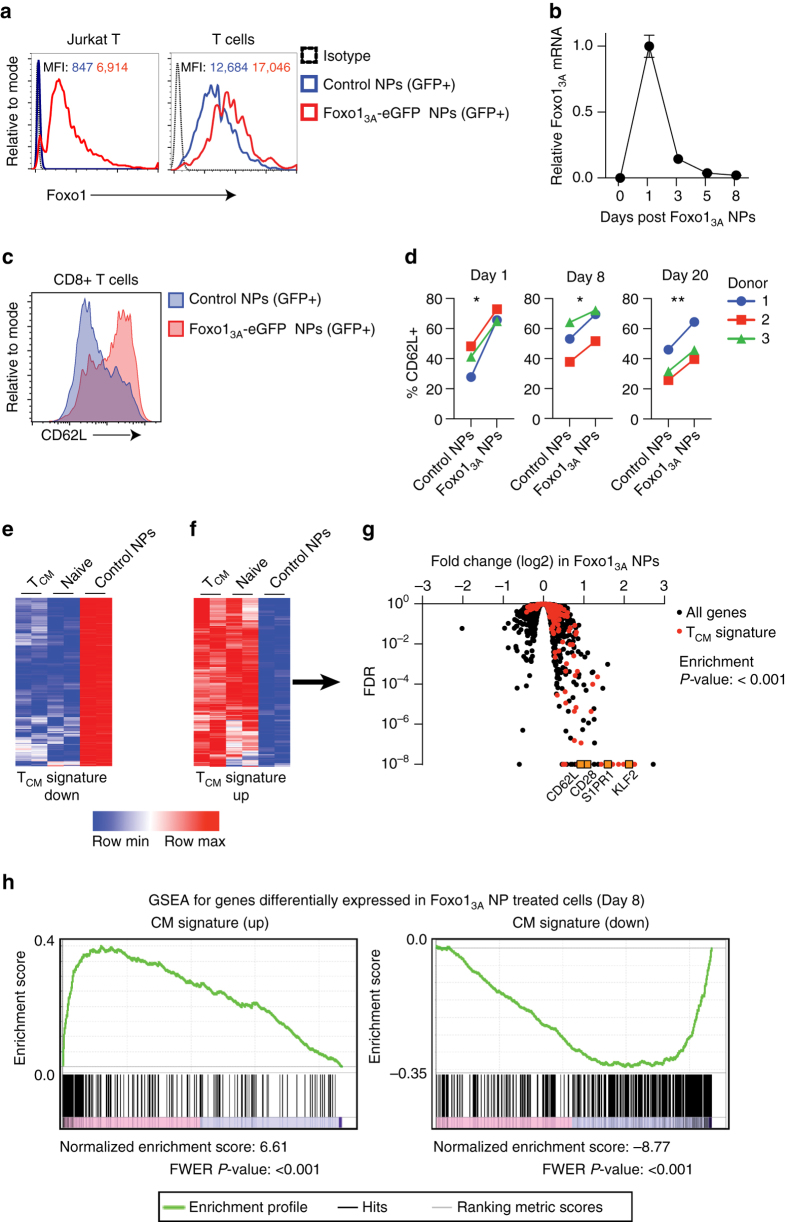
Nanoparticles can induce markers and transcriptional patterns characteristic of memory T-cells. **a** Expression of total Foxo1 protein measured by intracellular labeling in Jurkat and primary T-cells treated with CD3-targeted control (GFP+) or Foxo1_3A_-GFP NPs. Mean Foxo1 fluorescence intensities (MFI) for cells transfected with control NPs (shown in *blue*) compared to Foxo1_3A_-NPs (shown in *red*) are indicated above the respective histograms. **b** qPCR measurements of relative Foxo1_3A_ mRNA expression over time after cells were exposed to Foxo1_3A_-eGFP nanoparticles (NPs). Data are representative of two independent experiments. Graphs show mean ± SE. **c** Effect of CD8-targeted Foxo1_3A_-GFP NPs on CD62L expression after 24 h of particle treatment. **d** Percentage of CD62L^+^ cells in sorted CD8^+^ eGFP^+^ cells treated with CD8-targeting control or Foxo1_3A_/eGFP-encoding NPs at 1, 8, and 20 days of culture after the particles were introduced. These results are from three independent donors. *P < 0.05; **P < 0.01 between the indicated conditions as calculated from a ratio-paired *t*-test. **e**, **f** Heat maps of T_CM_ signature gene expression in T_CM_, naive, and control cells 8 days after treatment. **g** Volcano plot of differential gene expression in Foxo1_3A_-NP-treated cells after 8 days. T_CM_ signature genes and selected memory phenotype genes are indicated. *P* value of overlap between Foxo1_3A_ and the T_CM_ signature gene set was determined by GSEA (via analysis shown in **h**)

We next examined whether lymphocyte-targeted NPs can improve the therapeutic activity of CAR-T-cells by delivering mRNAs that program them toward a favorable phenotype. Clinical testing has already established that lymphocytes derived from CD62L^+^ central memory T-cells (T_CM_) present improved engraftment and function in animal models, and high fractions of CD62L^+^ T_CM_ cells in infused products are linked to successful CAR treatment^33, 34^. However, to develop into therapeutically relevant numbers, these cells must undergo multiple rounds of in vitro stimulation/expansion—a process that drives cells away from the T_CM_ lineage and toward terminal differentiation and senescence^35^. To address this problem, we manufactured T-cell-targeted NPs loaded with mRNA encoding the forkhead family transcription factor Foxo1, which controls the effector-to-memory transition in CD8 T-cells^22, 23^. During in vitro stimulation/expansion, TCR and cytokine signaling activate AKT kinase, which phosphorylates Foxo1 and thereby causes its cytoplasmic segregation and blocks its transcriptional activity. To maintain active Foxo1 levels in cultured T-cells, we used an AKT-insensitive nuclear retaining Foxo1_3A_ variant in which three key phosphorylated residues are mutated to alanine (Supplementary Fig. 5)^36^. We hypothesized that addition of Foxo1_3A_-containing NPs to T cell culture medium during ex vivo expansion would promote the development of CD62L + T_CM_ cells that have improved therapeutic potential. The effect transcription factors have on reprogramming is sensitive to the magnitude and duration of their expression. To determine these values after Foxo1_3A_-NP addition, we measured Foxo1 protein and mRNA expression in the treated cells. In the Jurkat T-cell line, endogenous expression of Foxo1 is low and Foxo1_3A_-NP treatment led to large increases in total expression of the factor, as measured by intracellular labeling (Fig. 4a, left panel). In primary T-cells, expression levels of Foxo1 protein is already high, and Foxo1_3A_-NPs only led to modest increases. These findings indicate that NP treatment can maintain near-physiological levels of the active transcription factor (Fig. 4a, right panel). To examine mRNA dynamics after it was delivered into activated, proliferating T-cells by NPs, we measured the expression of Foxo1_3A_ using real-time quantitative PCR specific for the engineered mRNA. We found mRNA expression was maximal at day 1, and was close to baseline by 8 days post-transfection (Fig. 4b). Treatment with Foxo1_3A_-NPs after T-cell priming in vitro rapidly increased expression of CD62L (Fig. 4c), which is the primary surface marker that distinguishes T_CM_ from effector and effector memory populations^37^. To determine if transient expression of Foxo1_3A_ can lead to persistent alterations in CD8^+^ T-cell differentiation, cells from three independent donors were treated with CD8-targeted Foxo1_3A_-eGFP NPs, sorted based on eGFP expression, and maintained in vitro. An increased frequency of CD62L^+^ cells was observed by 24 h after transfection, and this was maintained even 8 and 20 days after NP addition (Fig. 4d). Foxo1_3A_ overexpression did not induce T-memory stem cells, as the lymphocytes were universally CD45RO^+^ (Supplementary Fig. 6).

To understand the genetic regulatory network induced by Foxo1_3A_ and its connections to the T_CM_ lineage, we performed RNASeq on ex vivo-isolated naive CD8, T_CM_ CD8, and in vitro-cultured CD8 T-cells that were treated with Foxo1_3A_-encoding NPs or control particles. We identified a T_CM_ signature consisting of the most frequent 500 genes that have lower (Fig. 4e) or higher (Fig. 4f) expression in T_CM_ versus average CD8 T-cells. The majority of these genes are coordinately regulated in naive CD8 T-cells, which is consistent with the close transcriptional relationship we observed between naive and T_CM_ cells^38^. Foxo1_3A_-encoding NP treatments led to differential expression of many genes. As expected, these included those encoding the key memory transcriptional effector KLF2, and the surface molecules SELL (CD62L), CD28, and S1PR1, all crucial mediators of CD8 T_CM_ trafficking and function (Fig. 4g)^39, 40^. Overlaying the T_CM_ gene signature onto the Foxo1_3A_ volcano plot reveals a strong concordance of transcripts: this means T_CM_ signature genes were up-regulated in Foxo1_3A_-programmed CD8 cells. Gene set enrichment analysis confirmed the strong connection between Foxo1_3A_-regulated transcripts and T_CM_-associated gene expression (Fig. 4h).

**Fig. 5:**
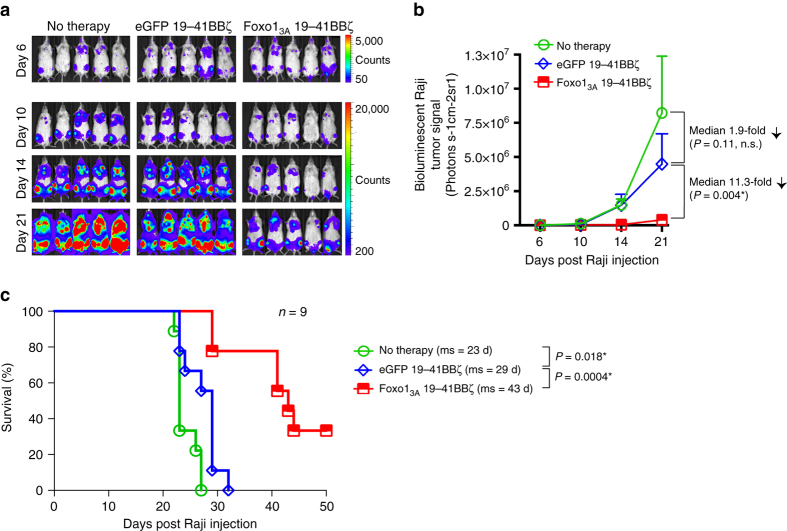
Foxo1_3A_-NP-transfection improves the anti-cancer activities of CAR T-cells. NSG mice were inoculated with CD19 + Raji-luc tumor cells. After 7 days the mice were injected with luciferin and imaged on an IVIS before being randomly sorted into groups (*n* = 9) with representative tumor burden. Next 2.5 × 10^6^ CD8+ 19-41BBζ CAR + T-cells (transfected with NPs loaded either with Foxo1_3A_ mRNA or GFP mRNA) were infused intravenously. Control mice received no treatment. **a** Representative IVIS imaging depicting five mice per cohort. **b** Quantified tumor burden (as mean radiance from luciferase activity from each mouse from **a** ± SE). Pairwise differences between groups were analyzed with the unpaired, two-tailed Student’s *t* Test; n.s., non-significant; *, significant. **c** Kaplan–Meier survival curves for treated and untreated control mice. Shown are nine mice per treatment group pooled from two independent experiments. ms, median survival. Statistical analysis between the treated experimental and the untreated control group was performed using the Log-rank test; *P* < 0.05 was considered significant

To test whether genetically imprinting a T_CM_ phenotype in CAR-T-cells translates into improved antitumor efficacy, we studied the effect of Foxo1_3A_-NP transfection on the in vivo activity of 19-41BBζ CAR-modified T-cells in a mouse model of B-cell lymphoma. Immunodeficient NOD.Cg-Prkdcscid Il2rgtm1Wjl/SzJ (NSG) mice were inoculated with 5 × 10^5^ CD19 + Raji cells expressing firefly luciferase. After 7 days, one treatment group received a single low-dose infusion of 2.5 × 10^6^ CD8 + 19-41BBζ CAR T-cells, the second group was injected with the same dose of Foxo1_3A_-NP transfected CAR T-cells, and controls received no treatment. After adoptive immunotherapy, we used bioluminescence imaging to serially quantify tumor growth and monitored overall survival. We found that low-dose treatments with conventional 19-41BBζ CAR-T-cells produced only a temporary delay in disease progression (29 compared with 23 days median overall survival in the untreated control group; Fig. 5a–c). By contrast, mice treated with Foxo1_3A_-transfected 19-41BBζ CAR-T-cells showed substantial disease regression, with an average 11.3-fold reduced tumor burden compared to 19-41BBζ CAR-T-cells 2 weeks after therapy (Fig. 5b), which translated into a median 14-day improvement in survival (Fig. 5c).

In summary, these results indicate that Foxo1_3A_-encoding NPs induce persistent alterations in surface markers, and transcriptional programming towards a T_CM_-like phenotype with improved antitumor function.

### Nanoparticle-mediated transfection can promote HSC expansion

We further examined the generality of our approach by genetically engineering stem cells, focusing on the problem that integration of HSCs into clinical use is limited by an inability to expand them ex vivo without inducing differentiation^24, 25^. Several stem cell self-renewal genes have been identified, including homeobox B4 (HOXB4) and Musashi-2 (MSI2)^26, 41^. Unfortunately, it is unsafe to permanently integrate these transgenes into the genome of human HSCs, as the factors they encode can promote malignant progression^42^.

**Fig. 6:**
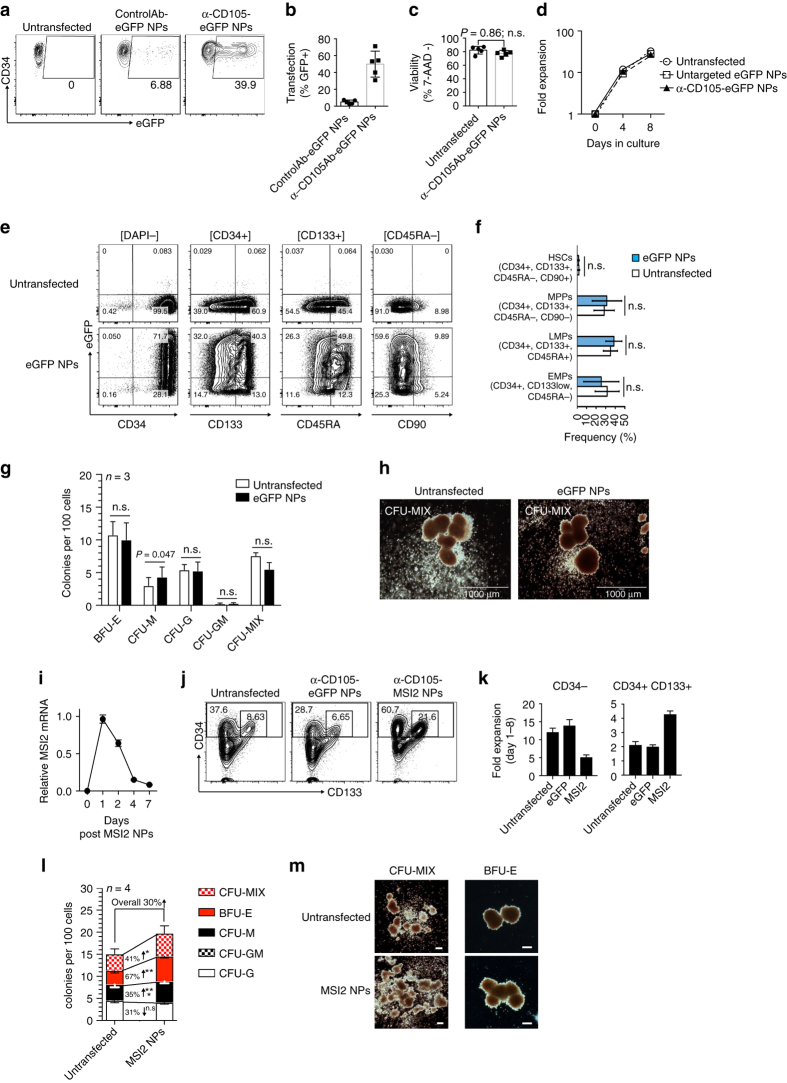
Transfection of stem cells with mRNA nanoparticles can promote their expansion and self-renewal. **a** Targeting of CD105 enables specific transfection of HSC CD34^+^ cells. Cells were left untreated, or transfected with eGFP-encoding mRNA in nanoparticles (NPs) coated with PGA coupled to a control antibody or anti-CD105. Transfection efficiency was assayed by flow cytometry after 24 h. **b** NP transfection efficiency in CD34^+^ samples from three independent donors. Viability is shown in **c**. **d** Expansion of CD34 + PBSCs after NP-transfection. **e** Phenotypical characterization of PBSC-derived CD34^+^ subpopulations after 2 days in culture. Cells were either transfected with eGFP NPs on day 1 or left unmodified. Gating is indicated in brackets on top of each column. **f** Summary *bar graph* showing mean frequencies and SE of primitive Hematopoietic Stem Cells (HSCs), Multipotent Progenitors (MPPs), Lymphoid-primed Multipotent Progenitors (LMPs), and Early Myeloid Progenitors (EMPs). PBSCs from four independent donors were analyzed. *Error bars* represent mean ± SE. **g** Colony output of sort-purified GFP-NP transfected versus unmodified CD34^+^ cells from day 7 cultures (*n* = 3 cultures from independent donors); n.s., non-significant. Arising colonies were identified as colony forming unit (CFU) granulocyte (CFU-G), macrophage (CFU-M), granulocyte-macrophage (CFU-GM) and burst forming unit-erythrocyte (BFU-E). Colonies consisting of erythroid and myeloid cells were scored as CFU-MIX; n.s., non-significant. *Error bars* represent mean ± SE. **h** Representative images of CFU-MIX colonies from untransfected and GFP-NP-transfected CD34^+^ cells (×4-magnification; *scale bar* 1000 µm). **i** qPCR measurements of NP-delivered Musashi-2 (MSI2) mRNA expression over time. *Error bars* represent mean ± SE. **j** Comparison of CD133 and CD34 expression in HSCs transfected with control GFP mRNA-NPs versus MSI2 mRNA-NPs, assessed by flow cytometry 8 days after NP exposure and cell expansion. Data represent two independent experiments conducted in triplicate. **k** Cellular fold expansion of CD34− (differentiated) and CD34^+^ CD133^+^ (progenitor) cells. *Bar graphs* show mean and SE of three independent experiments. Data represent two independent experiments conducted in triplicate. **l** Colony forming unit outputs of untransfected versus MSI2-NP-transfected HSCs (*n* = 3 cultures from independent donors); Pairwise differences between groups were analyzed with the unpaired, two-tailed Student’s *t* Test. *P = 0.049, **P = 0.012, ***P = 0.011; n.s., non-significant. *Error bars* represent mean ± SE. **m** Representative images of colonies from untransfected and MSI2-NP-transfected CD34^+^ cells (*scale bar*, 300 µm)

We first examined whether mRNA nanocarriers can perform specific transfection of CD34^+^ human HSCs without affecting viability or triggering differentiation. To accomplish this, we incubated CD34^+^ cells (that had been purified from cytokine-mobilized peripheral blood) with GFP mRNA-loaded NPs coated with a nonspecific antibody (control Ab-eGFP NPs) or anti-CD105 (α-CD105 NPs). We chose to target CD105 because this surface marker is specific for the most immature, long-term repopulating HSCs^43, 44^, and because it can mediate the internalization of bound material^45^. We found that α-CD105-coated NPs produced efficient transfection levels (mean 49% ± SE/6.9%; Fig. 6a, b; Supplementary Fig. 7), which were similar to those described for transductions of these cells using highly purified lentiviral vectors^45^. In contrast, transgene expression in HSCs treated with control NPs coated with nonspecific antibodies was low (mean 4.9% ± SE/0.9%). Importantly, NP-transfection did not affect viability or proliferative activity of expanded HSCs (Fig. 6c, d). We also could not measure phenotypic differences between NP-transfected and unmodified HSCs (Fig. 6e, f), which indicates that receptor-mediated internalization of bound NPs from the cell surface does not trigger undesirable differentiation of these hematopoietic progenitor cells. In line with these results, colony forming unit (CFU) assays revealed no difference in the ability of NP-transfected HSCs to form multi-lineage colonies when compared with unmodified HSCs (Fig. 6g, h).

To determine if transient expression of self-renewal genes directed by mRNA-carrying NPs can facilitate stem cell expansion, we loaded α-CD105-targeted NPs with synthetic mRNA encoding MSI2 and added them to cultures of HSCs. Using real-time quantitative PCR specific for the engineered (codon-optimized) MSI2 mRNA, we found that mRNA expression in HSCs was maximal at day 1, and was close to baseline by 7 days post-transfection (Fig. 6i). This transient NP-mediated upregulation of MSI2 increased the frequency and total numbers of CD34^+^ CD133^+^ cells by 2.6-fold and 2.3-fold, respectively, after 8 days of culture (Fig. 6j, k). At the same time, the outgrowth of differentiated CD34^−^ HSC populations was reduced by two-fold (Fig. 6k). To confirm that MSI2 NP-transfection enhances the regenerative potential of human HSCs, we examined their clonogenic potential in colony forming cell (CFC) assays. NP-mediated expression of MSI2 increased the overall ability of HSCs to form multi-lineage colonies by an average 30% (Figs. 6l, m), principally due to a 67% increase in erythroid burst-forming units (BFU-E) and a 41% increase in the most primitive CFU-granulocyte erythrocyte monocyte megakaryocyte (CFU-MIX) colony types.

In summary, these results demonstrate that mRNA NPs that induce key regulators of self-renewal can accelerate in vitro growth and regenerative potential of primitive HSCs.

## Discussion

As research findings produce new insights into the molecular mechanisms and master regulators that control cell fate and function, the list of genes potentially useful for rationally designing powerful disease-fighting cytoreagents is growing longer^46^. Unfortunately, the available gene therapy toolbox, which includes virus-mediated transduction approaches and physical electroporation methods, does not allow clinicians to apply the potential of genetic engineering without adding specialized protocols to an already complex and costly manufacturing process.

Here, we demonstrate using two therapeutic cell-based products–CAR-programmed T-cells and stem cells–that appropriately designed mRNA nanocarriers can transiently program gene expression to improve their therapeutic potential. We show how cell function and/or differentiation can be permanently reprogrammed by the simple addition of bioengineered NPs to cultures of cells used for therapy. This nanotechnology platform does not require special cell handling, so it can be easily integrated into established protocols for the manufacture of therapeutic cells without changing the workflow, or the equipment used in the process. Compared to RNA electroporation, which is currently the method of choice for ‘hit-and-run’ gene therapy in cell-based products^47–49^, this could provide a significant advantage in manufacturing. As depicted in Fig. 2, in addition to the expensive equipment involved, electroporation requires many culture medium exchanges, centrifugation steps, and washing cycles. Each of these procedures is prone to error, and increases the risk of contamination and compromising the final cell product. Even state-of-the-art flow electroporation devices reduce cell viability, and hence product yield and quality^50, 51^. Our approach does not rely on mechanical permeabilization of cell membranes to deliver transgenes. Instead, engineered NPs bind to target cells and stimulate receptor-mediated endocytosis – a physiological process that provides entry for the RNA they carry without compromising cell viability (Fig. 2).

Cell-penetrating peptides (CPPs), which are small proteins that facilitate cellular uptake of various molecular cargos, have also been used to transport therapeutics into cells^52^. Even large proteins that harbor CPP domains can be introduced into the cytoplasm using this approach. However, specific targeting of selected cell types is not possible with CPPs, and protein transfer is relatively inefficient^53, 54^. The duration of therapeutic impact also strongly depends on the half-life of the transferred protein. By contrast, nanocarriers loaded with synthetic mRNA are targeted to specified cells, and every RNA molecule they deliver serves as a template for the translation of multiple protein copies.

The surface molecules we targeted to deliver the NPs are just examples of the many antigens that could be used to selectively shuttle mRNA into cytoreagents. For instance, to selectively modify only defined T cell subsets, such as antigen-experienced lymphocytes, activation markers (e.g., CD25, 4-1BB, OX40, or CD40L) could be targeted. Similarly, antibodies recognizing CD34, CD133, or CD46 could be explored as targeting ligands for human HSCs. Also, the choices for the core polymer and the charge-negating coating material are flexible, and will likely be optimized before production in a clinical setting comes about. In terms of the former, our group tested a panel of cationic matrices, including hyperbranched STAR polymer, polyethylene glycol-grafted polyethylenimine, and mesoporous silica nanoparticles, and elected to use PBAE 447 based on its superior transfection efficacy and low cytotoxicity in primary cells. This compatibility is the result of the high biodegradability of this formulation, which has a half-life between 1 and 7 h in aqueous conditions^55^. That time frame is ideal for gene therapy, as the polymer condenses and protects the mRNA against degradation while it is encapsulated in the endosome, but releases it soon after transfer into the cytoplasm, thus enabling transcription of the encoded protein. Importantly, in all NP designs we tested, a negatively-charged coating was required to shield the positive charge of RNA/PBAE polyplexes and prevent off-target binding.

In summary, our project demonstrates that exposing pharmaceutical cells to an NP reagent can improve their therapeutic value by accomplishing ‘hit-and-run’ gene modification. This platform does not add complexity to manufacturing because it involves no special equipment or training. Thus, it can substantially streamline the manufacture of cell-based therapies at clinical scales, which means that treating patients with genetically engineered cells could become less expensive, and more effective as a disease-fighting intervention.

## Methods

### PBAE 447 synthesis

This polymer was synthesized using a method similar to that described by Mangraviti et al^55^. 1,4-butanediol diacrylate was combined with 4-amino-1-butanol in a 1.1:1 molar ratio of diacrylate to amine monomer. The mixture was heated to 90 °C with stirring for 24 h to produce acrylate-terminated poly(4-amino-1-butanol-co-1,4-butanediol diacrylate). 2.3 g of this polymer was dissolved in 2 ml tetrahydrofuran (THF). To form the piperazine-capped 447 polymer, 786 mg of 1-(3-aminopropyl)-4-methylpiperazine dissolved in 13 ml THF was added to the polymer/THF solution. The resulting mixture was stirred at RT for 2 h, then the capped polymer was precipitated with 5 volumes of diethyl ether. After the solvent was decanted, the polymer was washed with 2 volumes of fresh ether, then the residue was dried under vacuum for 2 days before it was formed into a stock of 100 mg/ml in DMSO, which was stored at −20 °C.

### PGA-antibody conjugation

15 kD poly-glutamic acid (from Alamanda Polymers) was dissolved in water to form 20 mg/ml and sonicated for 10 min. An equal volume of 4 mg/ml 1-ethyl-3-(3-dimethylaminopropyl) carbodiimide hydrochloride (Thermo Fisher) in water was added, and the solution was mixed for 5 min at RT. The resulting activated PGA was then combined with antibodies at a 4:1 molar ratio in phosphate buffered saline (PBS) and mixed for 6 h at RT. To remove unlinked PGA, the solution was exchanged 3 times against PBS across a 50,000 NMWCO membrane (Millipore). Antibody concentrations were determined using a NanoDrop 2000 spectrophotometer (Thermo Scientific). The antibodies we used for T cell experiments were anti-CD3 (clone OKT3, BioXCell; Cat# BE0001-2), anti-CD4 (clone OKT4, BioXCell; Cat# BE0003-2), anti-CD8 (clone OKT8, BioXCell; Cat# BE0004-2), and anti-CD28 (clone 9.3, BioXCell; Cat# BE0248). Clone C1.18.4 was used as a control antibody (BioXCell; Cat# BE0085). For HSC transduction, we used polyclonal goat anti-human CD105 (R&D Systems; Cat# AF1097) and non-specific polyclonal goat IgG antibodies (Biolegend; Cat# 400102). There were no dilutions for all above antibodies. We coupled them to polyglutamic acid at a 4:1 molar ratio.

### mRNA synthesis

Codon-optimized mRNA for eGFP, Foxo1, Trex2, TRAC-megaTAL, and Musashi-2, which were fully substituted with the modified ribonucleotides pseudouridine (Ψ) and 5-methylcytidine (m5C) and capped with ARCA, were produced by TriLink Biotechnologies. We conjugated Ψ and m5C-modified eGFP mRNA with cy5 (also from TriLink) to track delivery of these transcripts.

### Nanoparticle preparation

mRNA stocks were diluted to 100 µg/ml in 25 mM nuclease-free sodium acetate buffer, pH 5.2 (NaOAc). PBAE-447 polymer in DMSO was diluted to 6 mg/ml in NaOAc, and added to mRNA at a 60:1 (w:w) ratio. After the resulting mixture was vortexed for 15 s at medium speed, it was incubated for 5 min at room temperature so NPs could form. To add targeting elements to the NPs, PGA-linked antibodies were diluted to 250 µg/ml in NaOAc and added at a 2.5:1 (w:w) ratio to the mRNA. The resulting mixture was vortexed for 15 s at medium speed, and then incubated for 5 min at room temperature to permit binding of PGA-Ab to the NPs. The NPs were lyophilized by mixing them with 60 mg/ml D-sucrose as a cryoprotectant, and flash-freezing them in liquid nitrogen, before processing them in a FreeZone 2.5L Freeze Dry System (Labconco). The lyophilized NPs were stored at −80 °C until use. For application, lyophilized NPs were re-suspended in a volume of sterile water to restore their original concentration.

### Nanoparticle characterization

The hydrodynamic radius of the particles we created was measured with a Nanosite (Malvern), and their zeta potential was determined using dynamic light scattering detected with a Zetapals instrument (Brookhaven Instrument Corporation). The particles were diluted 1:400 (v/v) in PBS (pH 7.4) for size measurements, and 1:40 for zeta potential quantitation. For transmission electron microscopy, a 25-μl sample of NPs was applied to a glow discharge-activated 200 mesh carbon/formvar-coated copper grid. After 30 s, grids were touched sequentially to a drop of ½ Karnovsky’s fixative, a drop of 0.1 M cacodylate buffer, 8 drops of dH_2_O, and then a drop of 1% (w/v) filtered uranyl acetate. These samples were examined using a JEOL JEM-1400 transmission electron microscope (JEOL USA).

### Cell lines and culture media

K562-CD19 and control K562 cells were provided by Dr. Stanley Riddell (Fred Hutchinson Cancer Research Center). TM-LCL is a CD19^+^ EBV-transformed lymphoblastoid cell line that has been optimized to use as a feeder for T cell expansion^56^. The Jurkat-E6 T cell line was obtained from the American Type Culture Collection (Cat# TIB-152). These cells were cultured in T cell medium (TCM): RPMI-1640 containing 10% fetal bovine serum, 0.8 mM l-glutamine, 25 mM HEPES buffer, and 1% penicillin-streptomycin. Raji tumor cells (American Type Culture Collection; Cat# CCL-86), lentivirally transduced with firefly luciferase (Raji/ffluc cells^34^) were also cultured in TCM.

Cell lines were screened for the presence of mycoplasma on a monthly basis. Primary human peripheral blood mononuclear cells (PBMC) and T-cells were cultured in TCM supplemented with 50 IU IL-2/ml (Preprotech), or in ImmunoCult-XF T Cell Expansion Medium (XFSFM) (Stemcell) as indicated.

### mRNA transfection of T-cells

Cryopreserved PBMC from normal donors were thawed by drop-wise addition of warm TCM, followed by centrifugation (450 × *g* for 10 min). Where indicated, CD8 T-cells were isolated by negative selection (Stemcell). Cells were cultured in TCM + IL-2 at 10^6^ cells/ml and stimulated with CD3/CD28 beads (Dynabeads, Life Technologies) at a 1:1 bead:cell ratio. For experiments involving αCD3-targeted NP transfection, these beads were removed 24 h before NP addition.

For NP-mediated transfections, the T-cells were resuspended in XFSFM to a concentration of 2 × 10^6^/ml. Antibody-targeted NPs containing 2.5 µg of mRNA/10^6^ cells were mixed into this suspension for an exposure of 2 h at 37 °C before diluting it four-times by adding T cell medium supplemented with 50 IU IL-2/ml. Control NPs contained eGFP mRNA. TCRα gene editing NPs contained TRAC-megaTAL, Trex2, and eGFP mRNAs at a 42:42:16 w:w:w ratio. Foxo1_3A_-NPs contained Foxo1_3A_ and eGFP mRNAs at an 84:16 w:w ratio.

For electroporation, 2 × 10^6^ T-cells were washed twice with PBS containing 0.5% bovine serum albumin (BSA), resuspended in 100 µl of T-cell electroporation medium (Lonza) containing 3 µg of eGFP mRNA, transferred to an electroporation cuvette, and treated in a Nucleofector (Lonza) instrument using program T-20. The electroporated cells were transferred into a plate containing 2 ml TCM + IL-2 without antibiotics.

In all T-cell genome editing experiments, cells were incubated at 30 °C for 40 h following nanoparticle transfection. This transient cold shock was previously reported to enhance genome editing in mammalian cells^57^.

### Nanoparticle transfection of CD34^+^ cells

CD34^+^ cells purified from peripheral blood stem cells (PBSC) previously mobilized from normal donors were obtained from the Hematopoietic Cell Processing and Repository Core at the Fred Hutchinson Cancer Research Center. After thawing, the cells were counted then cultured overnight at a concentration of 10^6^/ml in HSC medium: StemSpan SFEMII serum-free medium supplemented with 50 ng/ml human Stem Cell Factor (Scf), 50 ng/ml murine Flt3/Flk-2 ligand, and 25 ng/ml human thrombopoietin (Stemcell Technologies). The next day the cells were harvested, counted, and resuspended in 100 µl HSC without cytokines at 2.5 × 10^4^ cells/well in a 96-well tissue culture plate (Costar). The cells were treated with CD105-targeted or control NPs containing 1 µg eGFP or Musashi-2 mRNA per well for 1 h, then washed twice with 1 ml HSC medium without cytokines. Washed cells were then transferred into 500 µl complete HSC medium in 24-well tissue culture plates; 48 h later, the cells were labeled for CD34, CD133, and CD105 (BioLegend) for analysis by flow cytometry.

### PCR amplification and detection of indels for TCRα

Indel detection was performed with a Geneart Genomic Cleavage Detection Kit (Invitrogen) according to the manufacturer’s instructions. Briefly, T-cells were lysed, and genomic DNA flanking the TCRα MegaTAL target site was amplified by PCR using these primers: TRAC-Forward CCCGTGTCATTCTCTGGACT, and TRAC-Reverse ATCACGAGCAGCTGGTTTCT. The PCR product was denatured, re-annealed, and treated with the detection enzyme. Indel formation was assessed by comparing gel band density for germline vs specifically cleaved bands.

### Lentiviral transduction and expansion of T-Cells using 19-41BBζ CAR

Human anti-CD19 CAR construct containing 41BB and CD3ζ signaling domains (19-41BBζ) was modified with a single StrepTag as described^58^ and transferred into the epHIV7 lentiviral vector. VSVG pseudotyped lentivirus was produced via calcium phosphate transfection (Invitrogen) of Lenti-X 293 T-cells (Clontech) with epHIV7 lentiviral vector and the viral packaging plasmids pCMVdR8.91 and pMD2.G. For lentiviral transduction, T-cells were transferred to retronectin-coated plates (Takara) with 8 µg/ml polybrene and 19-41BBζ-CAR encoding lentivirus at a MOI of 5:1, then spin infected for 1 h at 800 × *g* at 34 °C. For selective expansion of 19-41BBζ-transduced cells, the lymphocytes were stimulated with irradiated (7000 rads) CD19+ TM-LCL cells at a 1:7 ratio in TCM + IL-2.

To prepare tumor-targeting cells for in vivo therapy, CD8 T-cells were activated and transfected with α-CD8-targeted NPs containing eGFP or Foxo1_3A_ + eGFP mRNA on day 1 and 2 after activation. On day 3, GFP + cells were sorted from each treatment group, and transduced with 19-41BBζ-CAR-encoding lentivirus, followed by expansion for 5–7 days in TCM + IL-2. CAR T-cells were expanded with irradiated TM-LCLs. To maintain a differentiated state during this secondary expansion, T-cells were transfected with α-CD8-targeted NPs containing eGFP or Foxo1_3A_ + eGFP mRNA 1 day before and 1 day after addition of TM-LCL cells.

### Cell sorting and flow cytometry

Data were acquired using a BD LSRFortessa or FacsCanto II cell analyzer running FACSDIVA software, sorted on the BD FACS ARIA-II, and analyzed with FlowJo v10.1. Antibodies used in flow cytometry are listed in Supplementary Table 1.

### Cytokine secretion assays

T-cell IFN-γ release was measured with ELISA (R&D Systems) 48 h after stimulation by irradiated TM-LCL (a CD19^+^ EBV-transformed lymphoblastoid cell line that has been optimized for use as a feeder cell for T-cell culture)^59^ or C42 prostate control tumor cells as a negative control.

### Intracellular staining for foxo1

10^6^ Jurkat T-cells were transfected with anti-CD3-targeted NPs containing 3 µg eGFP mRNA, or 2.5 µg Foxo1_3A_ and 0.5 µg eGFP mRNA. 24 h later, cells were fixed with 4% paraformaldehyde in PBS, washed once, and permeabilized with 90% ice-cold methanol for 30 min. These samples were blocked with 0.5% BSA in PBS at room temperature, then labeled with rabbit anti-Foxo1 (clone C29H4) or isotype (clone DAE1), followed by anti-rabbit IgG F(aB′)2 Alexa-647 (Cell Signaling).

### CAR T cell killing assay

Specific cytolysis of CAR target cells was assayed by flow cytometry. Target K562-CD19 cells were labeled with low (0.4 µM), and control K562 with high (4.0 µM) carboxyfluorescein succinimidyl ester (CFSE) for 15 min at 37 °C. Both samples were washed in complete medium containing serum, mixed at a ratio of 1:1, then co-cultured with 19-41BBζ−transduced CAR T-cells at the indicated effector:target ratios. To assess specific cytolysis, each condition was stained with anti-CD8 mAbs (BioLegend; Cat# 301035, used at 1:200 dilution) to identify T-cells and with 7AAD to exclude dead cells, and analyzed by flow cytometry. Specific cell killing was assessed by measuring the ratio of viable CD19^+^ target cells (low CFSE) to control CD19- K562 cells (high CFSE).

### Microscopy

10^6^ T-cells in 400 µl of XFSFM were treated with anti-CD3-targeted NPs containing 3 µg cy5-labeled eGFP mRNA for 1 h at 4 °C for surface binding, followed by a 2-h incubation at 37 °C for internalization. Following these treatments, the cells were washed 3 times with cold PBS, and loaded onto poly-l-lysine (Sigma)-coated slides for 30 min at 4 °C. The samples were fixed in 2% paraformaldehyde, mounted in ProLong Gold Antifade reagent (Invitrogen), and imaged with a Zeiss LSM 780 NLO laser scanning confocal microscope.

### RNA isolation, qPCR, sequencing, and bioinformatic analysis

After cells (T-cells in Fig. 4b, HSCs in Fig. 6g) were lysed in Trizol reagent (Ambion), total RNA was isolated using a DirectZol kit (Zymo) with on-column DNA digestion following the manufacturer’s instructions. For real-time quantitative PCR (qPCR), cDNA was prepared with a high capacity cDNA kit (Applied Biosystems). Expression levels of codon-optimized (nanoparticle-delivered) FOXO1_3A_ (Fig. 4b) and codon-optimized MSI2 (Fig. 6i) relative to the housekeeping gene B2M were measured using PrimeTime qPCR assays (Integrated DNA technology) and a QuantStudio5 machine (Applied Biosystems). The following primers were used: Foxo1_3A_ Forward: GGACAGCCTAGAAAGAGCAG; Foxo1_3A_ Probe: AGGTCGGCGTAGCTCAGATTGC; Foxo1_3A_ Reverse: CTCTTGACCATCCACTCGTAG; MSI2 (codon optimized) Forward: CTGCTAGACCTGGCGGATT; MSI2 (codon optimized) Reverse: CCGTACAGATCGGCCACT. For RNAseq analysis, RNA samples were isolated from in vitro-cultured control NP- and Foxo13A-NP-treated CD8^+^ cells after 3 and 8 days, and compared with sorted reference naive (CD8^+^ CD45RA^+^ CD62L^+^ CCR7^+^) and T_CM_ (CD8^+^ CD45RA^−^ CD62L^+^ CCR7^+^) cells from two independent donor-matched cryopreserved PBMC samples.

RNASeq libraries were prepared using the TruSeq sample preparation kit (Illumina) according to the manufacturer’s instructions. Libraries were sequenced for 50 cycles (paired end) with a HiSeq platform (Illumina). We aligned results that passed Illumina’s base call and quality filters to the human hg38 genome using TopHat v2.1.0. Counts were generated for each gene with htseq-count (v0.6.1p1), implemented in the “intersection-strict” overlap mode. We used the GLM method in edgeR for data normalization and differential expression analysis. T_CM_ signature gene sets were defined as the top 500 genes ranked by statistical significance with higher (T_CM_ Up) or lower (T_CM_ Down) expression in CD8^+^ T_CM_ versus donor-matched control NP-treated CD8^+^ T-cells at day 8. Gene set enrichment was analyzed with GSEAPreranked software using gene lists ranked by the sign of the fold change × 1/(*P* value)^58^. Raw and processed data from RNAseq analysis have been deposited in NCBI’s Gene Expression Omnibus, GEO series accession number GSE89134^60^.

### Mice and in vivo tumor models

Animals were housed in the animal facility of Fred Hutchinson Cancer Research Center, and used in the context of an animal protocol approved by their Institutional Animal Care and Use Committee. Four- to 6-week-old female NOD.Cg-*Prkdc* ^*scid*^ *Il2rg* ^*tm1Wjl*^/SzJ (NSG) mice were bred in house and engrafted via tail vein with 5 × 10^5^ Raji cells expressing firefly luciferase. One week later, tumor burden was determined by bioluminescent imaging, and mice with detectable tumor were sorted into groups with matching tumor burden. Groups were then randomly assigned to treatment conditions, receiving no therapy, or intravenous injections of 2.5 × 10^6^ CD8+ 19-41BBζ CAR+ cells transfected with eGFP or Foxo1_3A_ mRNA encapsulating nanoparticles. Based on pilot studies, 5 mice per group were used for data analysis to provide an 80% power to attain a *P*-value of 0.02. The investigators were not blinded to group allocation.

### In vivo bioluminescence and fluorescence imaging

We used D-Luciferin (Xenogen) in PBS (15 mg/ml) as a substrate for F-luc (imaging of Raji-luc lymphoma cells). Bioluminescence images were collected with a Xenogen IVIS Spectrum Imaging System (Xenogen, Alameda, CA). Living Image software version 4.4 (Caliper Life Sciences) was used to acquire (and later quantitate) the data 10 min after intraperitoneal injection of D-luciferin into animals anesthetized with 150 mg/kg of 2% isoflurane (Forane, Baxter Healthcare). Acquisition times ranged from 10 s to 5 min.

### Colony-forming cell assay

For Colony-forming Cell (CFC) assays 400 sorted HSCs cells were seeded into 1 ml MethoCult H4435 (StemCell Technologies). Hematopoietic colonies were scored after 12–14 days. Arising colonies were identified as colony forming unit- (CFU-) granulocyte (CFU-G), macrophage (CFU-M), granulocyte-macrophage (CFU-GM) and burst forming unit-erythrocyte (BFU-E). Colonies consisting of erythroid and myeloid cells were scored as CFU-MIX.

### Statistical analysis

The statistical significance of the differences we measured in T-cell expansion (Fig. 2d, e), viability (Fig. 2g), lentiviral gene transfer (Fig. 3f), bioluminescent tumor signal (Fig. 5b), the viability of HSCs (Fig. 6d), and the HSCs differentiation (Fig. 6f, g, l) was analyzed with the unpaired, two-tailed Student’s *t* Test. The *P* value of overlap between Foxo1_3A_ and the T_CM_ signature gene set (Fig. 4e–g) was determined by GSEA (via analysis shown in Fig. 4h. A *P* value  < 0.05 was considered significant. We characterized survival data (Fig. 5c) using the Log-rank test. All statistical analyses were performed using GraphPad Prism software version 6.0.

### Study approval

Blood samples were obtained from healthy donors. Donors provided written informed consent for research protocols approved by the Institutional Review Board of the FHCRC. The FHCRC Institutional Care and Use Committee approved all mouse experiments.

### Data availability

Raw and processed data from RNAseq analysis have been deposited in NCBI’s Gene Expression Omnibus, GEO series accession number GSE89134. Step-by-step protocols describing nanoparticle synthesis, as well as T-cell transfection were uploaded to Protocol Exchange (“Stephan Lab-FHCRC”). All other relevant data can be requested from Dr M. Stephan by others both within and outside the scientific community.

## Electronic supplementary material

Supplementary Information
