# Supplementary material for: Hit-and-run programming of therapeutic cytoreagents using mRNA nanocarriers
Source: Nat Commun. 2017 Aug 30;8:389. doi: 10.1038/s41467-017-00505-8 (PMC5577173; doi:10.1038/s41467-017-00505-8)
Supplement: Supplementary file 1 — Supplementary Information [file 41467_2017_505_MOESM1_ESM.pdf]

File Name: Supplementary Information
Description: Supplementary Figures and Supplementary Table

File Name: Peer Review File

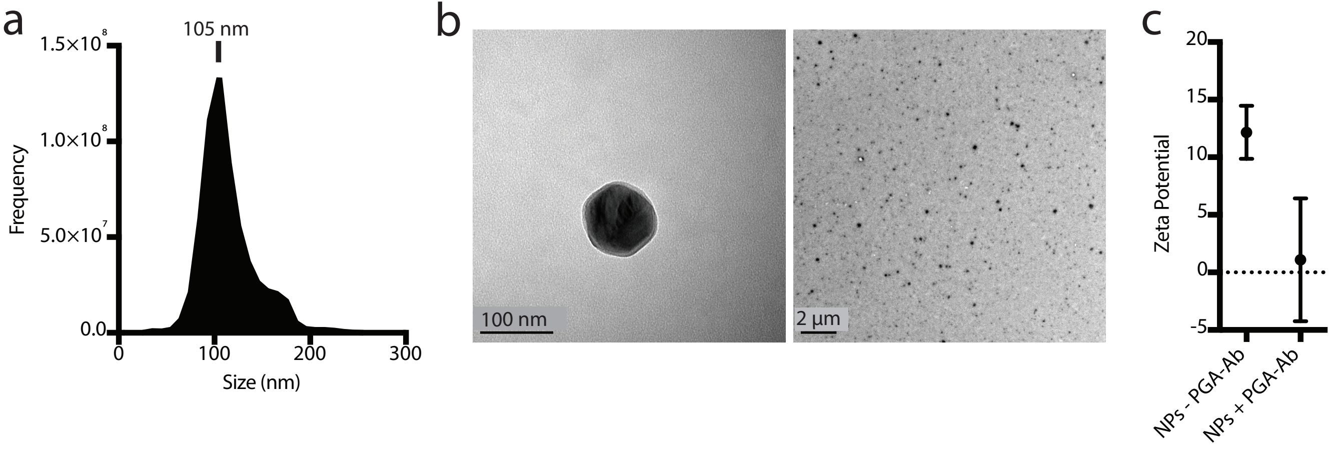

Supplementary Figure 1 Physical properties of mRNA-loaded nanoparticles (NPs). (**a**) Finite track length adjustment size distribution of individual particles. (**b**) Transmission electron microscopy of a single NP (left, scale bar = 100 nm) and a population of NPs (right, scale bar = 2 µm). (**c**) Zeta potential of control NPs (-PGA-Ab) compared with those coated with PGA coupled with antibodies (+PGA-Ab), measured after diluting them 1:40 in PBS pH 7.4 (n=5).

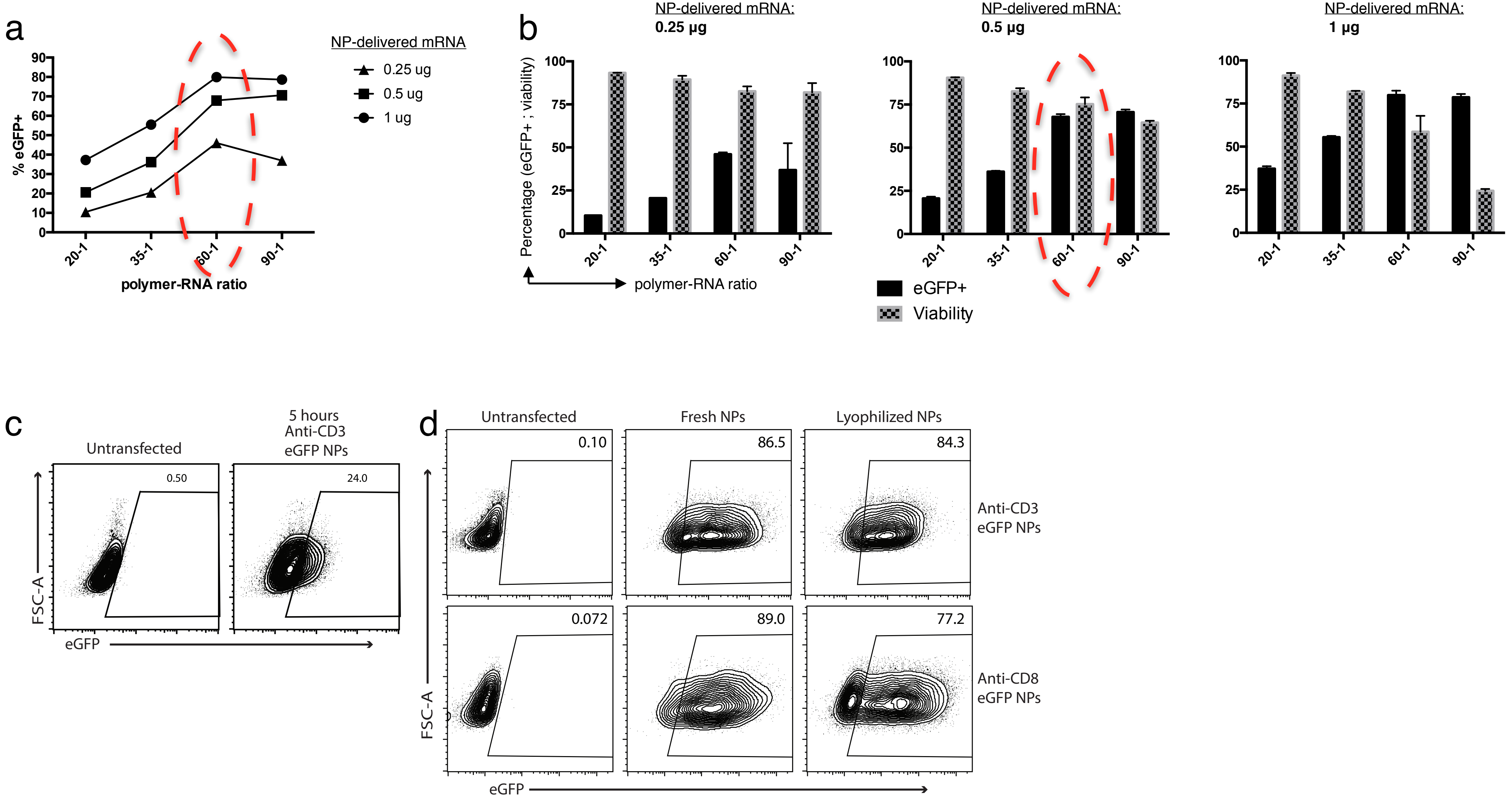

Supplementary Figure 2 Optimally formulated mRNA nNanoparticles (NPs) rapidly transfect human T cells without affecting viability. transfection is rapid and not affected by lyophilization. (**a, b**) In order to evaluate the *in vitro* gene transfer capability of T cell-targeted mRNA NPs, transfection assays were carried out at different polymer-mRNA ratios and various amounts of delivered mRNA (per 10^6 T cells). Maximum transfection in T cells was achieved with a polymer-mRNA ratio of 60-1. Best combined viability and transfection was observed when transfecting T cells with 0.5 µg mRNA at a 60-1 polymer-mRNA ratio, which is the formulation we chose for the experiments described here (encircled in red). (**c**) eGFP expression in 10^6 activated T cells that were either untreated, or measured 5 h after addition of eGFP-encoding NPs targeted with anti-CD3. (**d**) Transfection efficiency of NPs is maintained after lyophilization and resuspension. 10^6 activated T cells per condition were transfected with NPs (targeted via anti-CD3 or anti-CD8 antibodies) which were either prepared freshly, or lyophilized, stored at -80 °C, and then suspended to their original volume.

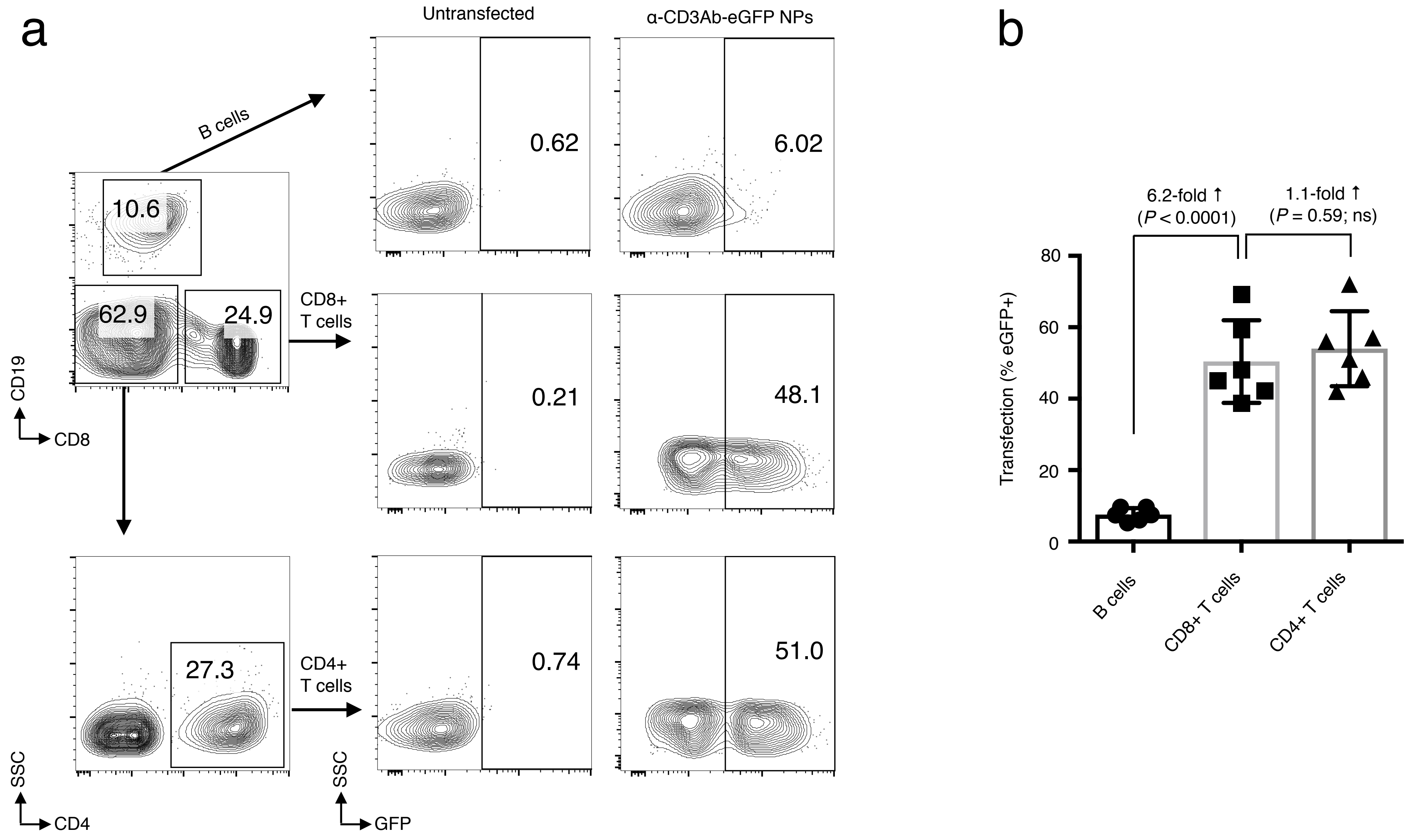

Supplementary Figure 3 CD3-mediated targeting confines nanoparticle interactions to T cells. Unstimulated bulk peripheral blood mononuclear cells (PBMC) were directly incubated with CD3-targeted NPs carrying GFP mRNA. One day later, we compared transfection efficiencies in T cells versus B cells. Flow cytometry plots are shown in (**a**). (**b**) Summary plot showing mean transfection efficiency and S.E.M. of three independent experiments conducted in duplicate.

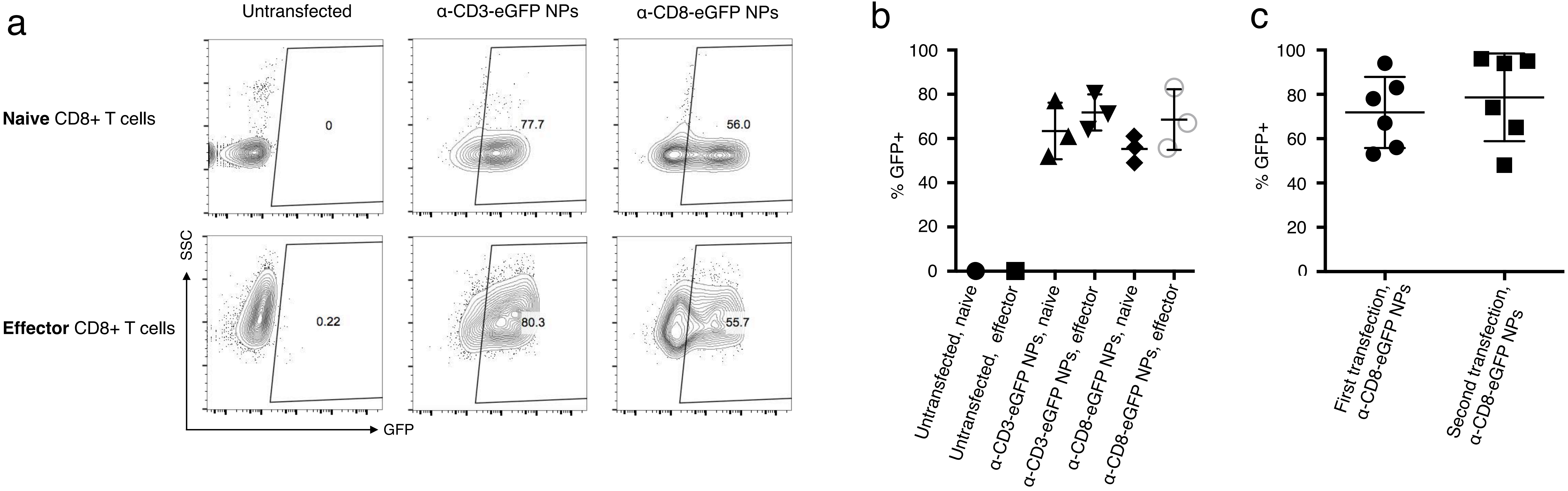

Supplementary Figure 4 Transfection of lymphocyte-targeted mRNA nanoparticles is unaffected by cell activation or proliferation. Isolated human CD8+ T cells were either left unstimulated or activated with beads coated with antibodies against CD3 and CD28. 10^6 T cells per condition were then transfected with NPs (targeted via anti-CD3 or anti-CD8 antibodies). (**a**) Flow cytometry of NP transfection efficiencies (based on eGFP signals) after 24 h. (**b**) Bar graphs show mean and S.E.M. of three independent T_cell donors. (**c**) In parallel experiments, we compared NP-mediated transfection of freshly activated versus restimulated T cells. Shown are mean transfections and S.E.M of three independent experiments conducted in duplicate.

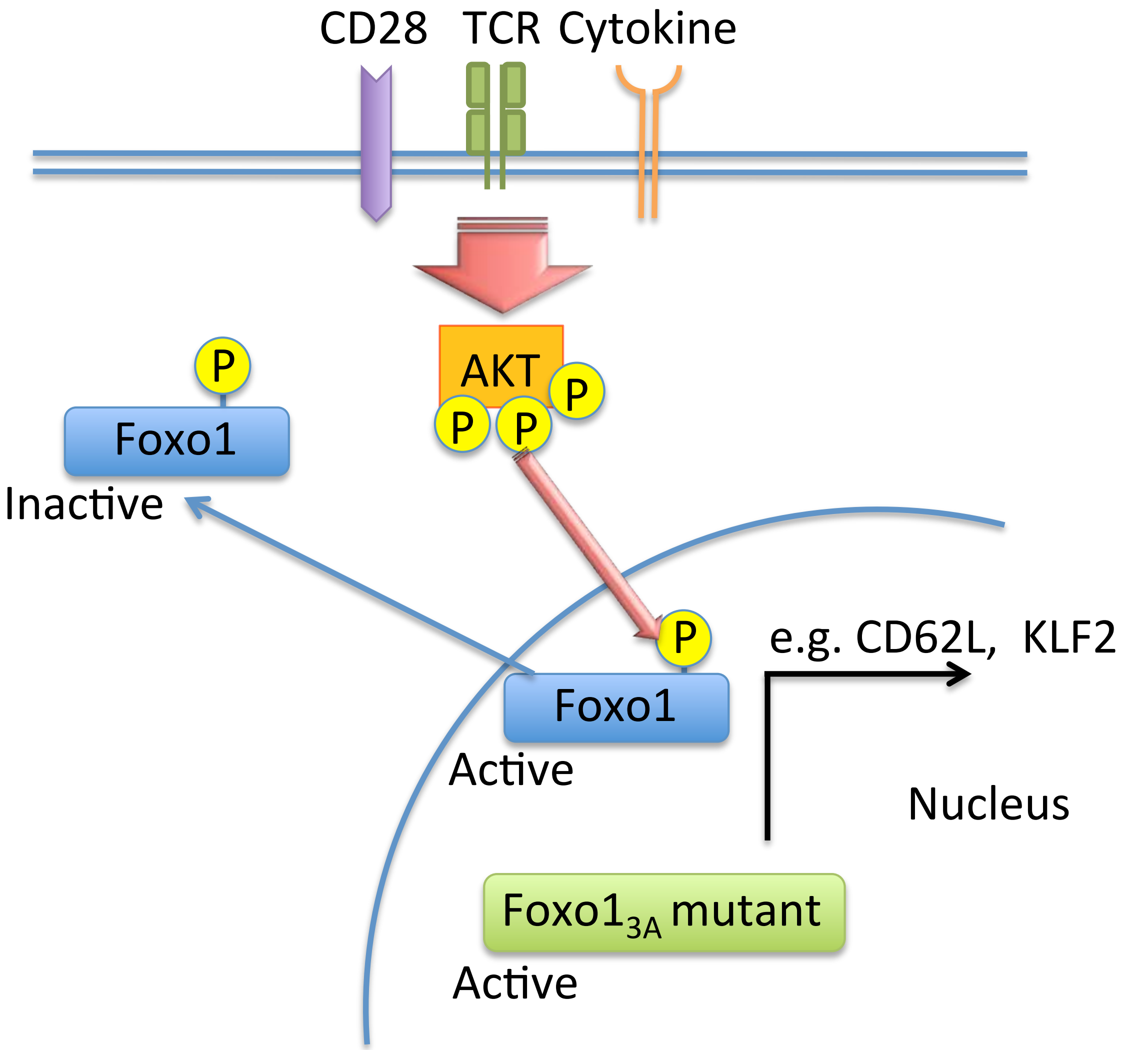

Supplementary Figure 5 Schematic illustrating regulation of wild-type Foxo1 versus the constitutively active nuclear retaining Foxo1$_{3A}$ variant in T cells. T cell stimulation activates PI3K/AKT signaling to phosphorylate Foxo1 leading to nuclear export and inactivation of transcriptional activity. In contrast, the Foxo1$_{3A}$ mutant has three alanine substitutions that inhibit AKT-mediated phosphorylation, which renders it insensitive to nuclear export[36].

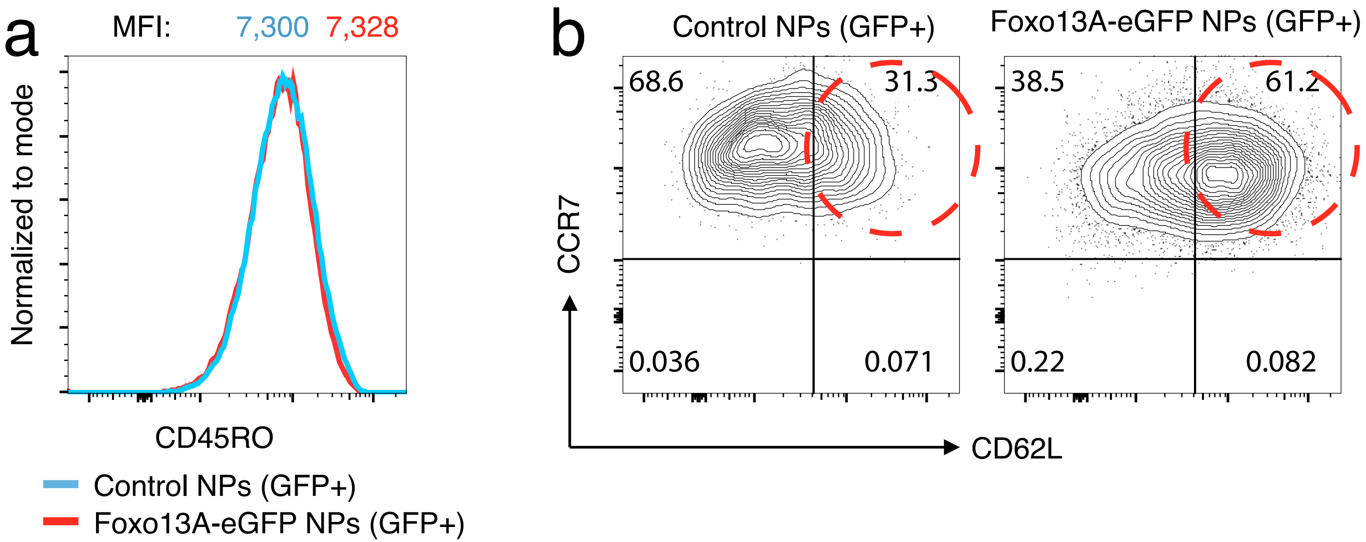

Supplementary Figure 6 Flow cytometric analysis of CD45RO (a), CD62L and CCR7 (b) expression levels on human CD8+ T cells transfected with CD8-targeted polymeric nanoparticles carrying control GFP mRNA or Foxo1$_{3A}$ mRNA. Following transfection, cells were expanded for 2 weeks before analysis. MFI, mean fluorescence intensity.

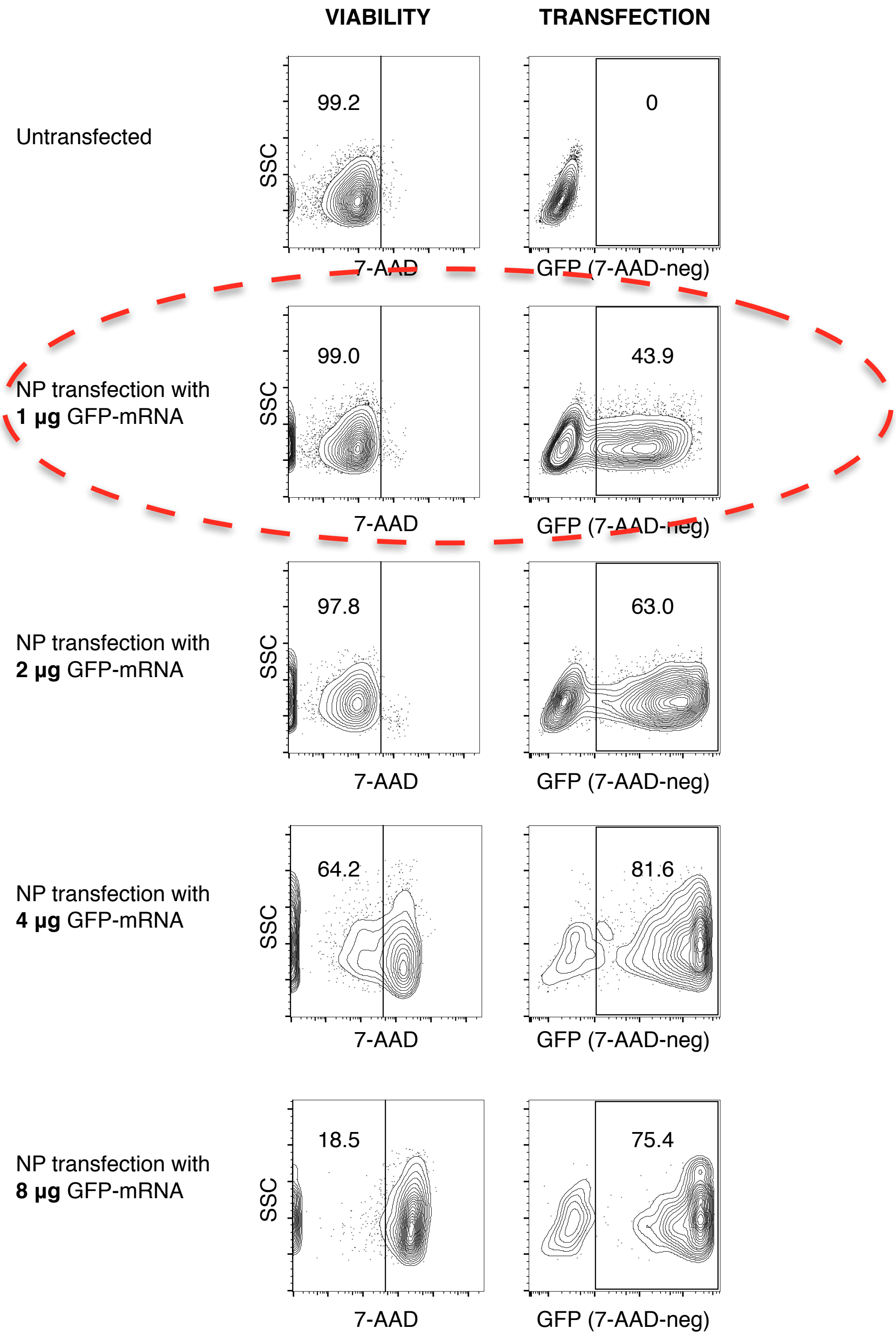

Supplementary Figure 7 Appropriately-dosed CD105-targeted mRNA nanoparticles efficiently transfect human hematopoietic stem cells (HSCs) without affecting their viability. 25×10^3 HSC CD34+ cells obtained from mobilized PBSCs were transfected with different doses of eGFP-encoding mRNA in nanoparticles (NPs) coated with PGA coupled to anti-CD105. Transfection efficiency and viability were assayed by flow cytometry 24 h after NP exposure. Based on these data, we chose to transfect HSCs at a dosage of 1 µg mRNA per 25×10^3 cells (encircled in red).

Supplementary Table 1. Antibodies used for flow cytometry

Antibody Specificity	Dye	Clone	Supplier
CD34	BV421	561	Biolegend
CD34	PECF594	563	BD Biosciences
CD90	APC	5E10	Biolegend
CD90	Pe-Cy7	5E10	BD Biosciences
CD105	PE-Cy7	43A3	Biolegend
CD49F	PE	GOH3	Biolegend
CD28	BV 510	CD28-2	Biolegend
CD62L	APC-CY7	DERG56	Biolegend
CD62L	PE-CY5	DERG56	Biolegend
CD45RA	ALEXA 700	HI1000	Biolegend
CD45RA	APC-Cy7	5H9	BD Biosciences
CCR7	PE	G043H7	Biolegend
CD3	APC	HIT3A	Biolegend
CD3	BV421	HIT3A	Biolegend
CD45R0	PERCP-CY5	UCHL1	Biolegend
CD8	BV421	SK1	Biolegend
IL2	BV421	MQ1-17H12	Biolegend
IFN-G	PE	B27	Biolegend
CD19	PE	H1B19	Biolegend
CD133	PE-Vio615	AC133	Miltenyi
CD133	PE	AC133	Miltenyi
Foxo1	--	C29H4	Cell Signal
anti-Rabbit IG FAB2	Alexa 647	--	Cell Signal
StrepTag II	biotin	--	--
Streptavidin	APC	--	ebioscience
7AAD	--	--	ebioscience
